# High performance of an enzyme linked immunosorbent assay for American tegumentary leishmaniasis diagnosis with *Leishmania (Viannia) braziliensis* amastigotes membrane crude antigens

**DOI:** 10.1371/journal.pone.0232829

**Published:** 2020-05-07

**Authors:** María E. Bracamonte, Agustín Moya Álvarez, Andrea M. Sosa, Carlos L. Hoyos, Juan J. Lauthier, Silvana P. Cajal, Marisa Juarez, Renato E. Uncos, Fernando J. Sánchez-Valdéz, Leonardo Acuña, Patricio Diosque, Miguel A. Basombrío, Julio R. Nasser, Yoshihisa Hashiguchi, Masataka Korenaga, Paola A. Barroso, Jorge D. Marco

**Affiliations:** 1 Facultad de Ciencias de la Salud, Instituto de Patología Experimental, Universidad Nacional de Salta / Consejo Nacional de Investigaciones Científicas y Técnicas, Salta, Argentina; 2 Department of Parasitology, Kochi Medical School, Kochi University, Nankoku, Kochi, Japan; 3 Instituto de Investigaciones en Enfermedades Tropicales (IIET), Sede Regional Orán, San Ramón de la Nueva Orán, Salta, Argentina; 4 Laboratorio de Química Biológica y Biología Molecular (LQByBM), Facultad de Ciencias Naturales, Universidad Nacional de Salta, Salta, Argentina; 5 Faculty of Health Sciences, Kochi Gakuen University, Kochi, Kochi, Japan; Rajendra Memorial Research Institute of Medical Sciences, INDIA

## Abstract

The diagnosis of American tegumentary leishmaniasis (ATL) still requires the design of more effective tools. *Leishmania (Viannia) braziliensis* is the causal agent of the 90% of Argentinean ATL cases. Considering the current knowledge, an ELISA based crude antigen (CA) for the diagnosis was designed. Ninety-nine subjects diagnosed as ATL, 27 as no-ATL, and 84 donors from non-ATL-endemic areas were included in this study. The current ATL diagnosis was based four techniques, dermal smear microscopic examination (parasitological test), PCR, Leishmanin skin test, and clinical records. We obtained CA extracts from promastigotes and amastigotes from macrophage cultures of different zymodemes of endemic *Leishmania* species circulating in the study area. Crude antigens from the ‘local’ main zymodeme of *L*. *(V*.*) braziliensis* showed the highest reactivity against anti-*Leishmania* antibodies compared to the other included species. The CA of amastigotes of this zymodeme was 3.4 fold more reactive than promastigotes one. Moreover, amastigote-membrane CA (MCA) were 3.6 fold more reactive than the soluble antigens. The MCA-ELISA reached a sensitivity and specificity of 98% (CI = 94.7%-100%) and 63.6% (53.9–73.1), respectively. When anti-*Trypanosoma cruzi* reactive sera were excluded, the specificity reached 98.4% (94.4–100), while the sensitivity was similar, with a positive predictive value (PV) of 98.6% (94.6–100) and negative PV of 96.3% (91.6–100). The performance of the MCA-ELISA results strongly contribute to the final diagnostic decision, since a non-reactive serological result almost discards the suspected ATL, because of its high negative PV. The developed MCA-ELISA showed a high diagnostic performance, which makes it a good candidate for ATL diagnosis, for seroprevalence studies, or for monitoring treatments efficacy.

## Introduction

Leishmaniasis is a group of diseases caused by various species of protozoa (*Kinetoplastida*: *Trypanosomatidae*) of the genus *Leishmania*. Its transmission occurs when a female phlebotomine sand fly infected with *Leishmania* spp. bites a potential host. The clinical manifestations depend mainly on the parasite species and the host’s genetic and immunological constitution [1, 2].

American tegumentary leishmaniasis (ATL) is an endemic disease in Argentina. The main etiological agent in the country is *Leishmania (Viannia) braziliensis*, with *L*. *(Leishmania) amazonensis* and *L*. *(V*.*) guyanensis* as the minor prevalent species in the endemic areas [3, 4]. In fact, more than 90% of the ATL cases have been caused by *L*. *(V*.*) braziliensis* [5]. The prevalence of this species in this areas was later confirmed by nested PCR and sequencing of cytochrome *b* (cyt b) gene [6].

The estimated incidence of ATL in Argentina reached 8.76 cases/year/10^6^ inhabitants, calculated from 1984 to 2005 case-reports [7], and 53.1% of the cases occurred in the north of Salta province. Since the diagnosis of ATL in the country depends mainly on the visualization of amastigotes in smears obtained directly from lesions, and other laboratory resources for confirming the cases are not always available, the incidence values might be underestimated or inaccurate. In addition, the time consuming microscopic technique (parasitological test) often shows low sensitivity and requires highly trained personnel [8]. The Leishmanin skin test (LST) is applied as complementary diagnostic test. However, it detects past infections or previous contact with the parasite, but not necessary an undergoing infection [4]. Furthermore, this geographic area is endemic for *Trypanosoma cruzi*, another member of the *Trypanosomatidae* family, which frequently shows cross reactivity with *Leishmania* spp. in different diagnosis techniques, leading to misdiagnosis, misinterpretation of epidemiological data, and to difficulties in disease treatment [4, 9].

Besides these methods, PCR has been an alternative approach to ATL diagnosis and *Leishmania* genus typing. In this way, a polymorphic specific-PCR (PS-PCR) approach developed and directly applied on clinical samples, and the sequencing of *cyt*b gene have shown promising results [5, 10]. However, these techniques are difficult to apply in rural health centers of endemic areas, because of the technical requirements and the relatively high cost, which still limit its routine use [11].

The diagnostic methods based on the detection of parasite specific antibodies have played an important role in the diagnosis of many parasitic diseases, mainly owing to the relatively low cost, high performance, high reproducibility, and low time consuming. In particular, nowadays ELISA is commonly used to diagnose visceral leishmaniasis (VL) and also has a higher sensitivity for ATL diagnosis than parasitological tests [12]. However, the general belief has been that serological assays for ATL/cutaneous leishmaniasis (CL) would be limited by low sensitivity, and consequently, they have not been considered for routine. Such a low diagnostic performance reported, could be related to lack of the optimized and/or standardized conditions of ELISA [13].

In this work, we analyzed the serological reactivity of different crude antigenic (CA) extracts of *Leishmania* spp. in order to develop an ELISA method suitable for the diagnosis of ATL. They were selected based on the current epidemiological, biological, and molecular information on the parasites of the genus *Leishmania* in the current study areas.

## Materials and methods

### Subjects and diagnosis of American tegumentary leishmaniasis

One hundred and twenty six patients with cutaneous or mucocutaneous lesions suspected of leishmaniasis were included in this study. They were recruited between 2000 and 2014. The diagnosis of ATL was performed in several institutions located in Salta province, Argentina by a criterion consisting in a parallel combination of methods previously described [5]. Briefly, they consist on the search for *Leishmania* amastigotes on smears of dermal scrapings, PS-PCR, LST, and the analysis of clinical features of the patients.

The anamnesis, biological sampling for parasitological and molecular diagnosis of ATL, and LST, were performed as previously described [5]. In addition, approximately five mL of peripheral blood were aseptically taken by puncture-aspiration of the antebraquial vein. Serum was separated by centrifugation at 3500 rpm for 10 min., and kept at -20°C until use for the serological reaction.

All patients diagnosed in the present work as ATL cases were systemically treated with 10–20 mg d^−1^ kg body wt^−1^ of pentavalent antimony over 25–30 days. In the cases of incomplete clinical cure, another treatment-cycle of the same extension with antimony or amphotericin B was given. Treatments and clinical follow-up were conducted by local physicians at the different medical institutions in Salta, Argentina, under the patients’ informed consent [5].

In addition, 40 sera from Chagas disease patients without tegumentary lesions were included in the study. They were recruited in the same period mentioned previously. Those patients inhabited in an endemic area of Argentina for this pathology. They were reactive for two serological methods, a recombinant ELISA and an indirect hemaglutination test (IHA) for the detection of anti-*T*. *cruzi* antibodies, as it was described previously [14].

**Fig 1:**
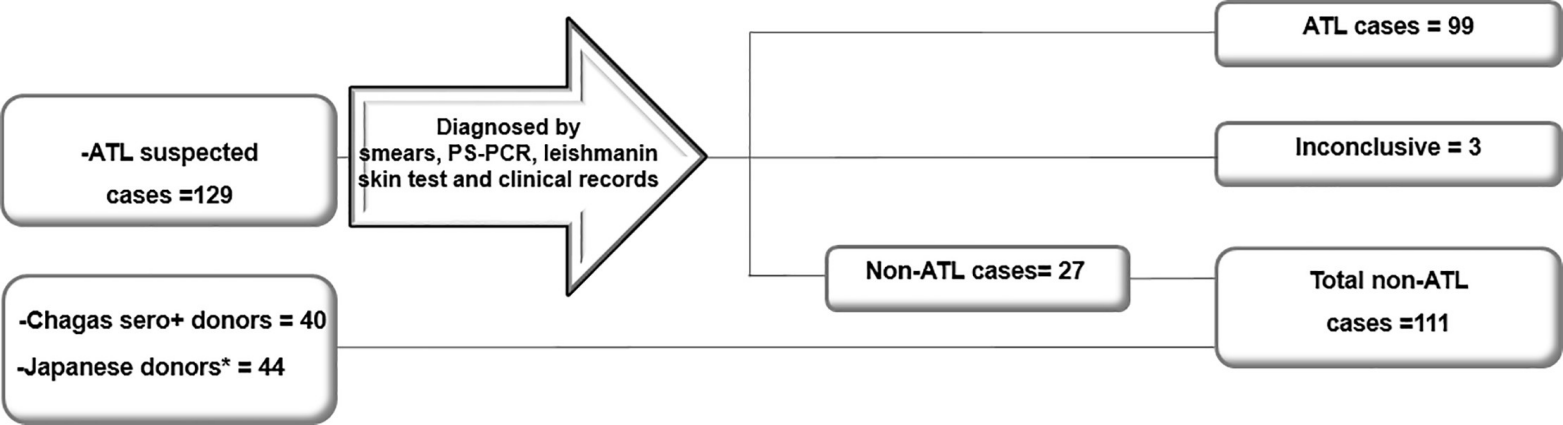
Groups of patients included in this study. It was defined the cases or non-cases of American Tegumentary Leishmaniasis (ATL) from suspected patients by four diagnostic methods mentioned above. Three of those 129 subjects were inconclusive and could not be grouped. Additionally, for the non-ATL cases, there were included two groups of donors without ATL suspicion: Chagas seropositives and Japanese donors from non-endemic geographic area for ATL and Chagas disease.

The set of samples was completed with 44 sera of healthy donors from Japan, a non-endemic area for either leishmaniasis or Chagas disease. These samples were kindly given by the diagnosis laboratory of Kochi Medical School Hospital on 2007. (Fig 1).

### Parasites and antigenic extracts

Three Argentinean and two World Health Organization (WHO) reference strains of *Leishmania*, previously characterized by multilocus enzyme electrophoresis, were included in this study (Table 1). Mass cultures of promastigotes of each strain were obtained, harvested and frozen following the procedures described previously [3].

**Table 1:** *Leishmania* zymodemes used for the preparation of antigenic crude extracts.

Strain designation	Species	Zymodeme	Cell stage
*Argentinean strains*			
MHOM/AR/02/OLO1	*L*. *(V*.*) braz*	KMS1	Amastigote, promastigote
MHOM/AR/02/CFO5	*L*. *(V*.*) braz*	KMS2	Promastigote
MHOM/AR/99/JDM1	*L*. *(V*.*) guy*	KMS4	Promastigote
*Reference strains*			
MHOM/BR/75/M2904	*L*. *(V*.*) braz*	KMS7	Promastigote
MHOM/BR/73/M2269	*L*. *(L*.*) amaz*	KMS6	Promastigote

*L. (V.) braz*: *Leishmania (Viannia) braziliensis*; *L. (V.) guy*: *L. (V.) guyanensis*; *L. (L.) amaz*: *L. (Leishmania) amazonensis*.

Intracellular amastigotes of *L*. *(V*.*) braziliensis* (MHOM/AR/02/OLO1) corresponding to KMS1, the main Argentinean zymodeme, were isolated from J774.1 cell line (RCB No. 0434. Riken Bio Resource Center, Saitama, Japan). Briefly, macrophages were cultured with complete RPMI 1640 medium (Nissui Pharmaceutical Co. Ltd, Japan) supplemented with 10% of heat-inactivated fetal bovine serum, 200 mM of L-glutamine, 50 mg/mL of streptomycin and 100 U/mL of penicillin, and placed at 37°C in a 5% CO_2_ 95% air mixture. Cells were infected with metacyclic promastigotes at a ratio of 10 / 1 (parasites / macrophage) and incubated in culture flasks at 34°C in a 5% CO_2_ 95% air mixture. The intracellular parasites were released from the host cells by three shake cycles in a vortex mixer followed by centrifugation at 3500 rpm for 10 min. Finally, the amastigotes were separated from the debris with 28% of Percoll (Sigma, St. Louis, USA) in phosphate-buffered saline (PBS), layered on 1 mL of 100% Percoll and centrifuged at 7000 rpm for 30 min at 48°C [15].

Soluble and membrane-enriched antigens were extracted from the promastigote and amastigote pellets following the procedures described previouslywith slight modifications [16]. Briefly, the pellets were resuspended in cold 0.05 M Tris-HCL, pH 7.8, in the presence of protease inhibitors (Roche, Mannheim, Germany). The cells were disrupted by sonication. After centrifugation the supernatant, named soluble crude antigen (SCA), was collected and the precipitate was treated with detergent/enzyme inhibitor mixture (1:1, v/v; 1.0% sodium dodecyl sulfate) for 12 hr at 4°C and centrifuged to remove final cellular debris. This supernatant was named membrane crude antigen (MCA). All of them were diluted with cold 0.05 M Tris, pH 7.8 to 10 mg of protein/mL [17]. Fractions of SCA and of MCA were pooled, and all the preparations were stored at– 70°C until use.

### Enzyme-linked immunosorbent assay procedures

The procedure was slightly modified from that described previously [18]. Briefly, the plates were sensitized overnight at 4°C with 100 μL of the SCA or MCA diluted in Bicarbonate/carbonate coating buffer, pH 9.6 at different concentrations, between 9 and 0.04 μg/mL of total protein. The optimal antigens concentration selected for each experiment is shown in the results section. Unbound antigen was removed by washing the wells three times with washing buffer, 0.05% Tween 20 in 0.01 M phosphate buffered saline, pH 7.0 (PBST). Two-hundred μL of Blocking One reagent solution (Nacalaitesque, Kyoto, Japan) were used in order to block non-specific binding reactions, following the manufacturer’s instructions. Serum samples were diluted between 1/20 and 1/4860 in a solution of 1/20 of Blocking One reagent in PBS, 0.1% Tween 20. The optimal serum concentration selected for each experiment is shown in the results section. One hundred μL of sera solution were added in each well and the plates were incubated at 37°C for one hour. After washing five times with PBST, 100 μL of goat-anti-human polyvalent Immunoglobulins (G, A, M)−Peroxidase conjugate (Sigma, St. Louis, USA), diluted 1/500 in the same solution used in the previous step, were added to each well and the plates were incubated at 37°C for one hour. Substrate and stop solution were prepared by dissolving the OPD (o-Phenylenediamine Dihydrochloride) tablets and diluting H_2_SO_4_ respectively, following the manufacturer’s instructions (Dako, Glostrup, Denmark). Absorbances (ODs) were read by a spectrophotometer at 490 nm.

For detection of anti-*T*.*cruzi* antibodies, recombinant 3.0 Chagatest ELISA (Wiener Lab, Rosario, Argentina) was applied to all sera included in the study by following maker's procedures indications. Under those conditions, the sensitivity (SE) and specificity (SP) reported reached 99.3% and 98.7% respectively. The resultant reactive sera were tested by IHA (Wiener Lab, Rosario, Argentina) and informed to local physicians, as it was described previously [14].

### Data analysis

#### *Leishmania* crude extracts avidity for anti-*Leishmania* antibodies

The ODs values obtained from ELISAs for comparisons of crude antigens (CA) from different zymodemes/species of *Leishmania*, amastigotes versus promastigotes, or SCA versus MCA were tabulated with Office Excel 2007 software (Microsoft Corporation, CA), and then analyzed with GraphPad Prism 5.0 (GraphPad Software Inc., CA) as follows. First, D´Agostino-Pearson normality test was applied to each data set. According with its results, the Wilcoxon signed rank test, or an ANOVA with Dunn´s multiple comparison was applied, depending on the number of groups compared. A *p*-value less than 0.05 was considered significant.

#### Diagnostic performance of ELISA with the selected crude antigens

The diagnostic performance of the tests was assessed by calculating several statistical indicators, including SE, SP and predictive values (PV). First the patients were classified into two groups: ATL cases and non-ATL cases based on the presence or absence of the disease, diagnosed based on combination of methods, as it was described above (Fig 1). In order to dichotomize the ELISA resulting ODs, a cut off value (CO) was established with the average of negative controls by the formula: ${\bar{OD}}_{neg}+6\times {SD}_{neg}$. An indetermination area of ODs was consequently defined, as the interval between *CO*±10% *CO*. For relativization proposes, the formula ${\bar{OD}}_{x}/CO$ was applied, were ${\bar{OD}}_{x}$ is the average of duplicate or triplicate ODs for each sample tested. The indicators were estimated applying Epidat 3.1 software. The PVs for the laboratory were calculatedby applying the Theorem of Bayes assuming a prevalenceof 54.82% for the year 2005.The proportions SE, SP and PVs were compared by Statistic-z analysis, using the same software [5, 19]. In addition, a Receiver Operator Curve (ROC) analyze was performed applying the GraphPad Prism 5.0 software, GraphPad Software Inc., San Diego, CA.

### Ethics statement

All patients and donors voluntarily consented to participate anonymously in this study. The procedures and the informed consent forms, signed by the subjects, were approved by the Bioethics Committee of the Medical College of Salta and thethe Bioethics Committee of the Health Ministry of Salta, Argentina following the Declaration of Helsinki Principles. The last reevaluation of projects including diagnosis of leishmaniasis was approved on 19^th^ March, 2015.

## Results

### Antigen selection

A series of experiments were designed in order to find the most suitable *Leishmania* crude antigenic extracts, according to their avidity for anti-*Leishmania* antibodies. For this purpose, a group of 17 to 22 sera, depending on each series experiment, from ATL-non Chagasic cases described in Fig 1 was studied in the same ELISA plate in order to maintain constant all the experimental conditions. Previously, the component concentrations and the conditions of each reaction assay were precisely adjusted by double-titration assays [20]. Since none of the data sets of the experiments passed the normality test, non-parametric tests were used for statistical significance estimation.

#### Antigens from ‘local’ *L*. *(V*.*) braziliensis* zymodemes have the highest avidity for anti-*Leishmania* antibodies

**Fig 2:**
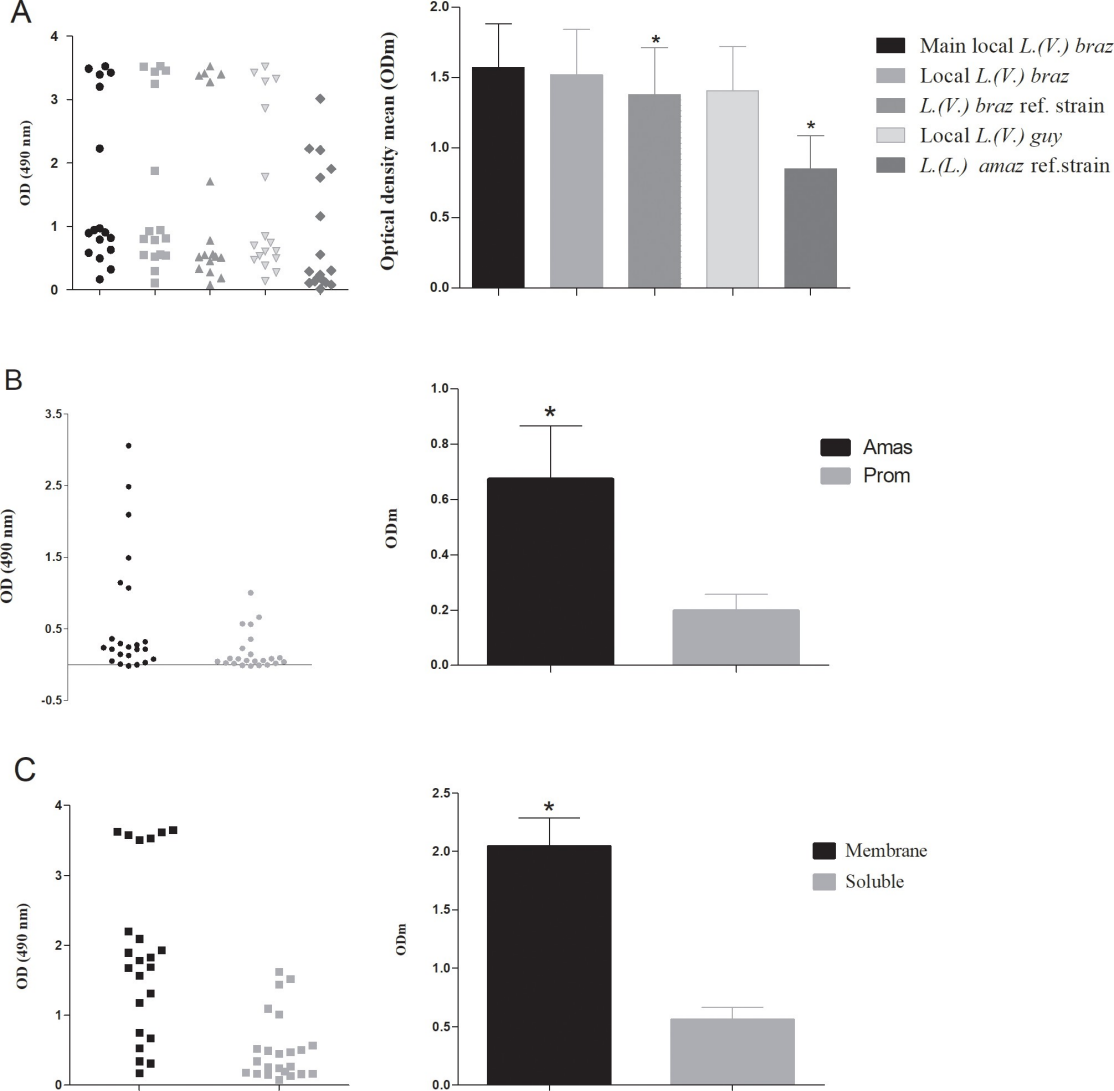
Comparison between the main local species, promastigoste vs. amastigote extracts and membrane based vs. soluble extracts. Comparison between Optical Density (OD) for each ATL case and resultant absorbance means (ODm) obtained from the following three consecutive assays, showing the differences among the avidity for anti-*Leishmania* antibodies. A) The ODs of 17 ATL cases and its ODm obtained by ELISA using promastigote Ags of five *Leishmania* zymodemes (0.15 μg/well), sera (1/2000,100 μl/well), and conjugate (1/500) conditions [*p by ANOVA/Dunn´s multiple comparison test vs. main local *L*. *(V*.*) braz*]; B) The ODs of 21 ATL cases obtained by using amastigote or promastigote Ags from Argentinean *L*. *(V*.*) braziliensis* main zymodeme (0.15 μg/well), sera (1/2000,100 μl/well), and conjugate (1/500) [*p by Wilcoxon test]; C) The ODs of 21 ATL cases obtained by using membrane or soluble extracts from amastigotes of Argentinean *L*. *(V*.*) braziliensis* main zymodeme under conditions previously described [*p by Wilcoxon test]. A p-value less than 0.05 was considered significant.

In the first assay of the series, 17 sera from ATL-non Chagasic cases were tested against crude antigenic extracts that were prepared from promastigotes cultures of the five zymodemes, corresponding to the three species previously incriminated as the causal agents of ATL in Argentina (Table 1) [3]. The ODs, ODs averages, and their statistical significance (p < 0.05) are shown in Fig 2A. The resulting ODs of the sera from non-ATL patients used as negative controls were similar to those from the blank one (data not shown). The SCA plus MCA extracted from the zymodeme KMS1 of *L*. *(V*.*) braziliensis*, the prevalent species in this endemic area, resulted in an average absorbance 1.85 fold higher than zymodeme KMS6 of *L*. *(L*.*) amazonensis*, which represents the minority species described in the area (Table 1) [4].

#### Amastigote antigens are more efficient than promastigote ones in the anti-*Leishmania* antibodies capture

In the next assay of the series, the efficiency of anti-*Leishmania* Igs capture between the different parasite cell stages was compared using 21 sera of ATL cases. To assess this assay, antigens from intracellular amastigotes and promastigotes were obtained from *L*. *(V*.*) braziliensis* KMS1 zymodeme expressing-strain, showing the highest avidity for anti-*Leishmania* Igs in the previous assay. The amastigotes CA extract was 3.4 fold more efficient than promastigote one in the anti*-Leishmania*Igs captured (Fig 2B).

#### The amastigote-membrane extracts are more reactive than the soluble ones

The assays described above were performed by using a mix of SCA and MCA following the study reported previously [15]. In this trial, the antigenic efficiency of both fractions was tested separately by using 21 sera from ATL cases by ELISA. The MCA were 3.6 fold more reactive than the SCA (Fig 2C). Then, the MCA was selected for its calibration and estimation of the diagnostic performance of ATL.

#### Diagnostic performance of an ELISA for ATL with amastigote-membrane antigenic extracts of *Leishmania (Viannia) braziliensis*

**Fig 3:**
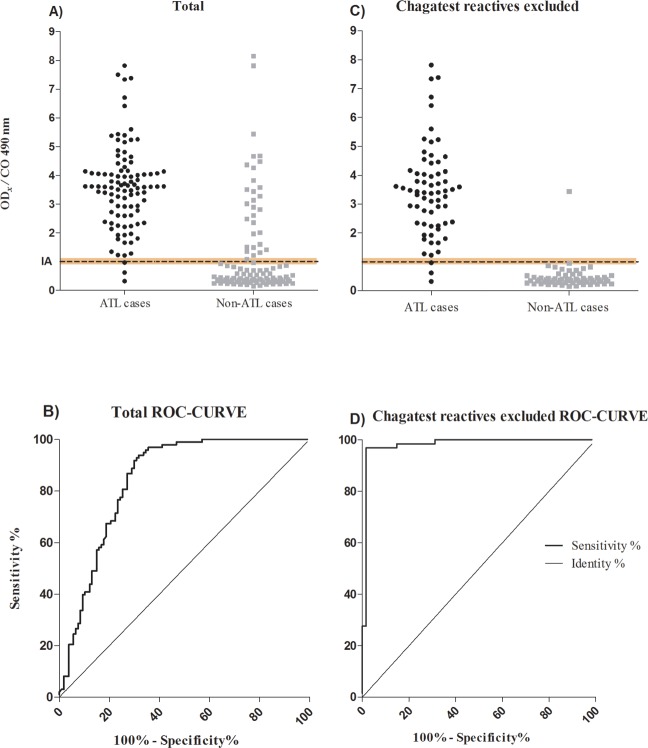
Diagnostic performance of the selected antigenic extract. Enzyme Lynked Immunosorbent Assay reactivity using the membrane amastigote extract against ATL and non-ATL cases sera **A**and **B**, and excluding Chagatest reactive samples, **C** and **D**. The relation ${\bar{OD}}_{x}$ / CO, where ${\bar{OD}}_{x}$ is the average of duplicate or triplicate ODs for each sample tested, and CO = Cut-off. The indetermination area (IA) is shown in dotted line, and defined as CO / CO±10%CO / CO = 0,9–1,1. **B** and **D** show the Receiver Operating Characteristic (ROC) Curves for ELISAs.

Once the MCA extract of *L*. *(V*.*) braziliensis* amastigotes was selected, the optimal concentration ranges of the antigen, sera, and conjugates were determined by double titration assays. To assess this, pools of 10 sera from patients with presence or absence of ATL were used in the assays. Then, preliminary ELISA tests were carried out with 45 sera from ATL or non-ATL groups in the same plate, at the following optimal concentrations defined: [MCA] = 0.1 μg/well, [Sera] = 1/200, and [conjugate] = 1/500. A serum from a patient with ATL was selected as positive, and six sera from non-ATL cases as negative controls respectively for the following determinations. For dichotomization, a CO and an indetermination area was established. Thus, the ODs determined for each patient serum, located at this range, cannot be classified as reactive or non-reactive by the ELISA employed, excluding the result of any diagnostic interpretation (Fig 3A and 3C).

The statistical indicators of diagnostics performance of the ELISA were estimated including sera from 98 ATL cases and 107 non-ATL cases (Fig 1, Table 2). The ODs resulting of the remaining five cases sera, 2.4%, were in the indetermination zone of the method, so they were excluded from the indicators estimation. In addition, detection of anti-*T*.*cruzi* antibodies was done on all sera of both group with recombinant Chagatest ELISA 3.0, a sensitive and specific commercially available test (Fig 3A and 3C).

**Table 2:** The performance of ELISA using amastigote-membrane extracts of ‘local’ *L*. *(V*.*) braziliensis* as antigens.

ELISA	Sensitivity	Specificity	PPV	NPV	No. of cases°	
	Percent (CI, 95%)				ATL	NoATL
Total	98 (94.7–100)	63.6 (53.9–73.1)	76.5 (63.1–79.1)	96.3 (92.5–100)	98	107
Chagatest reactives excluded	96.9 (92–100)	98.4 (94.4–100)*	98.6 (94.6–100)*	96.3 (91.6–100)	65	61

PPV/NPV: Positive/negative predictive values. CI: Confidence intervals. ELISA conditions: Ag: 0.1 μg/well; sera (1/200,100 μl/well); conjugate (1/500) (*p < 0.05 vs. total specificity and total PPV)

The present ELISA procedure showed high SE and negative PV, and low SP and positive PV. Nevertheless, they significantly increase when the reactive Chagatest ELISA patients were excluded from the analysis (Table 2). The areas under the ROC curve were 0.84; *p*< 0.0001, and 0.98; *p* <0.0001 showing the same effects of this exclusion (Fig 3B and 3D).

## Discussion

Serological methods are important diagnostic tools for several infectious diseases due to their low cost and technical simplicity. In this work, an ELISA for ATL diagnosis has been designed based on the current knowledge of the disease in endemic areas of Argentina. Thus, *L*. *(V*.*) braziliensis* has been incriminated as the causal agent of 90% of ATL cases [5]. The first assay proves that the avidity for the anti-*Leishmania* antibodies of the S plus MCA differs with the *Leishmania* species from which they were extracted. Thus, those CA obtained from local zymodemes of the prevalent species showed the highest avidity. In this sense, it is consistent also with the phylogenetic relationships of the genus *Leishmania*, as *L*. *(V*.*) braziliensis* is the most avid, followed by a species from the same subgenus, *L*. *(V*.*) guyanensis*. Accordingly, the S plus MCA from *L*. *(L*.*) amazonensis*, the most divergent LTA causal agents in the study area, reached almost a half of those maximum avidities (Fig 2A) [6, 21]. These differences on the species-specific epitopes avidity against anti-*Leishmania* antibodies was also observed in a serological study in Brazil, where CA from *L*.*(V*.*) braziliensis* had more diagnostic power compared to *L*. *(L*.*) amazonensis* one [22]. These differences could be attributed to a divergence at the proteome more than at the genome level, due to the complex post-transcriptional and post-translational regulation mechanisms of *Leishmania* genes [23, 24]. Thus, despite the few genome differences among species [25], there is a broad phenotype disease, and a great spectrum of specific antigenic epitopes.

The amastigotes S plus MCA showed more than three times reactivity than the promastigotes one (Fig 2B). The intracellular amastigote is the replicant-infective form of *Leishmania* present in the mammalian host. The promastigote stage occurs only at the beginning of the infection, until it is phagocytized by macrophagic-monocytic system cells [26]. Therefore, the humoral immune response would be triggered, mainly on the antigens expressed by the amastigote stage. However, further research is needed for understanding the bases for this antigenic behavior.

In addition, the amastigote MCA resulted almost four folds more reactive than the SCA, the highest difference observed in this work (Fig 2C). This result could be interpreted as follows; the membrane antigenic extract contains solubilized proteins with hydrophobic domains, necessary for their biochemical interaction with membranes. These epitopes are more immunogenic than the soluble ones, increasing the avidity for anti-*Leishmania* antibodies in an *in vitro* test.

The most frequently used method to diagnose ATL at the public health systems in endemic countries are the amastigote screening in smears of lesions and LST. However, both methods have some intrinsic limitations: Amastigotes detection in the smears is invasive and has a low sensitivity, particularly in ATL cases with chronic lesions, in wichthe number of parasites is extremely low and became difficult to demonstrate the presence in the lesions [27]. While LST does not distinguish between present and past infections, and thus its importance as a diagnostic tool is questionable for people living in endemic areas [12]. In this context, the advantages in diagnostic performance of the MCA included a higher sensitivity than the PS-PCR, dermal smears, or the *parallel*-combination ones, tested independently and reported in a previous study [5]. This serological method also resulted more sensible than those proposed for diagnosis of ATL so far. They are mainly based in CA extracts from promastigotes of *Leishmania* subgenus species, applied in areas where *Viannia* subgenus is prevalent [28]. Such a good good performance is also reflected in this work by the high negative PV.

Regarding to specificity, it is only compromised by the cross reaction with the anti-*T*. *cruzi* antibodies, since when the Chagasic patients are excluded from the analysis, it reaches to 98.4% (Table 2, Fig 3). Therefore, it was not affected by other endemic affections, or clinically comparable to ATL cutaneous diseases, represented in the non-ATL group tested (Fig 3).

Anti-*Leishmania* antibody detection in sera of patients also provides an independent and complementary result to the methods performed with material from the ATL lesions. This fact is relevant in cases when it is difficult to collect appropriated biological material from the lesions, because of their anatomical location, as in the case of secondary mucosal lesions, or because the sample extraction is painful due to a bacterial secondary infection.

Thus, the ELISA results influence the final medical decision, since a non-reactive one almost discard the suspected ATL, because of its high negative predictive value, (Table 2). In case of a reactive result, it indicates the presence of the disease only in non-Chagasic patients or in non-endemics areas of Chagas disease.

In conclusion, the current CA-based ELISA has demonstrated to be a method of high diagnostic performance, which makes it as good candidate for the diagnosis, seroprevalence studies, or for monitoring treatments of ATL, and design of defined antigens in further studies. However, in endemic areas of Chagas disease, the ATLdiagnosis has to be combined with serodiagnosis for the correct interpretation of the results.
